# Anthropogenic effects are associated with a lower persistence of marine food webs

**DOI:** 10.1038/ncomms10737

**Published:** 2016-02-12

**Authors:** Luis J. Gilarranz, Camilo Mora, Jordi Bascompte

**Affiliations:** 1 Department of Integrative Ecology, Estación Biológica de Doñana, (EBD-CSIC), Calle Américo Vespucio s/n, E-41092 Sevilla, Spain; 2 Department of Geography, University of Hawaii, Honolulu, Hawaii 96822, USA

## Abstract

Marine coastal ecosystems are among the most exposed to global environmental change, with reported effects on species biomass, species richness and length of trophic chains. By combining a biologically informed food-web model with information on anthropogenic influences in 701 sites across the Caribbean region, we show that fishing effort, human density and thermal stress anomaly are associated with a decrease in local food-web persistence. The conservation status of the site, in turn, is associated with an increase in food-web persistence. Some of these associations are explained through effects on food-web structure and total community biomass. Our results unveil a hidden footprint of human activities. Even when food webs may seem healthy in terms of the presence and abundance of their constituent species, they may be losing the capacity to withstand further environmental degradation.

## Abstract (web summary)

Human activity is affecting the diversity and abundance of marine organisms. Here, Gilarranz *et al*. show that the persistence of marine food webs is reduced by the effects of fishing pressure, human density, and thermal stress.

## Graphical abstract

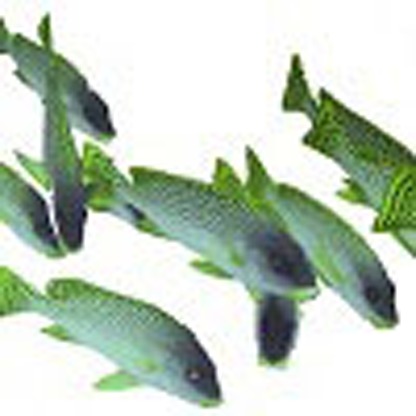

Anthropogenic influences are affecting species richness, abundance and genetic composition, with far reaching consequences for ecosystem functioning^1^,^2^,^3^,^4^. The majority of published studies, however, tend to treat species as independent units. In contrast, recent research is putting the emphasis on the consequences of global environmental change on the network of interactions among species^5^,^6^. Indeed, network persistence can be eroded before there is a change in community composition, with potential implications for systemic collapse^7^,^8^.

Previous work on trophic cascades has already identified anthropogenic signatures in food-web dynamics following the removal of key species, mainly top predators^9^,^10^,^11^,^12^,^13^,^14^,^15^. Here we address whether changes in food-web persistence can precede changes in species composition following anthropogenic influences. We tackle this problem by integrating several layers of information. Our starting point is a previously compiled regional marine food web encompassing 249 species/trophic groups and 3,313 interactions^16^, originated from reports on trophic interactions^17^ and stomach content analyses^18^. Next, we use a spatial dataset with species' demographic information from 701 locations across the Caribbean Sea, Gulf of Mexico and North Atlantic Ocean reefs (Fig. 1). Combining both data sets, we obtain not only the potential food web at every location, but also the body mass of the individuals, and the strength of the interactions.

Each local food web determines the skeleton of a dynamic model parametrized with empirical data (Methods). We estimate persistence as the fraction of species surviving at the end of a simulation^19^,^20^,^21^. We proceed by comparing the persistence of a local community with indicators of human influence at such a location, specifically fishing effort, nearby human density, thermal stress anomaly (TSA)—related with climate change, and the protected status of the area—protected versus non-protected as well as the size and age of the protected area. We also take in consideration recorded properties of the local communities, such as the total biomass or the architecture of each local food web—in terms of its tendency to be organized in compartments, where species within a compartment tend to interact among themselves while showing much fewer trophic links with species from other compartments. This later property seems to be pervasive across ecological networks^22^,^23^,^24^ and has been suggested to increase their robustness through the buffering of perturbations within specific compartments^21^,^25^.

Our results show that anthropogenic influences such as fishing affect the persistence of food webs beyond their more evident effects on species richness or total biomass. This suggests that human activities may have a previously unnoticed effect on natural communities by eroding their resilience in the face of further perturbations.

## Results

### Ecological associations

**Figure 1:**
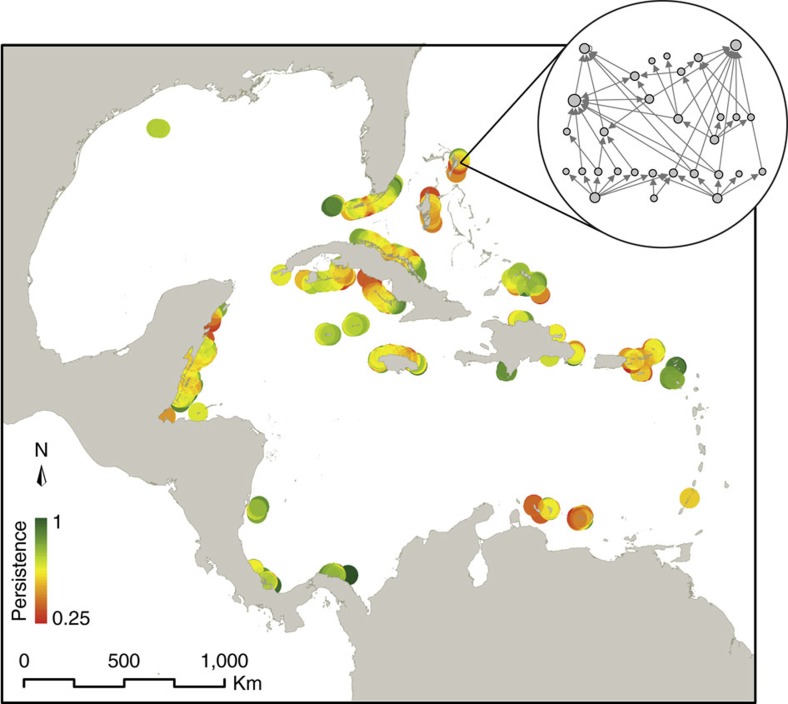
Persistence map of the Caribbean coral reef food webs. Each point indicates a sampling site and the colour represents the average persistence value of its simulated food web. Red and green colours represent low and high persistence, respectively. This map was made creating a 50 km buffer around each sampling site. The value of persistence of the sampling site is assigned to the area inside the buffer. Then, for representation purposes, each pixel in the map represents the average of the values of the buffers that overlap in that pixel. The insert shows one of the local food webs, where node size is proportional to its number of trophic interactions.

**Figure 2:**
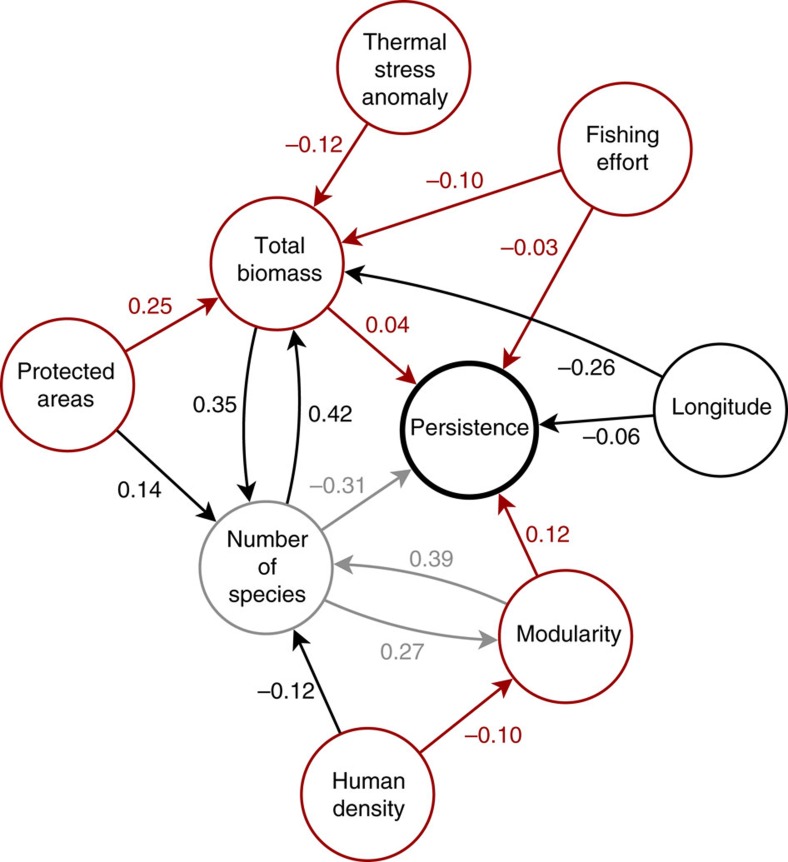
Network of statistical associations between variables. Each link represents a statistically significant effect of one variable on another. The numbers next to each link are the effect sizes. The associations between anthropogenic effects and food-web persistence are represented in red. Light grey represent already expected relationships from a theoretical standpoint.

Figure 1 represents a regional persistence map for the marine food web. This map provides the opportunity to look for signatures of food-web erosion along the wide range of human pressures existing in this region. First, and to fully disentangle the anthropogenic effects, we describe basic associations between ecological variables. Thus, we find the already known negative relationship between the number of species in a network and the number of surviving species at the end of the numerical simulations (Fig. 2; Supplementary Table 1). The larger the number of species, the narrower the parameter space in which species can coexist. This results in a lower persistence in larger food webs. We also find a significant relationship between the observed total biomass in one place and the number of species in that place (Fig. 2; Supplementary Table 2), probably mediated by trait complementarity^26^,^27^. These associations have to be taken into account when assessing the anthropogenic footprints on marine food webs. Next, we look at such anthropogenic effects across the regional persistence map.

### Anthropogenic associations with species richness and biomass

We observe that while fishing shows no association with the number of species present at a site, human density is negatively associated with species richness (Fig. 2; Supplementary Table 3). Arguably, this is because while fishing reduces species biomass, human density has an effect on the habitat inhabited by the local community, specially in water quality. This leads to a different species assembly near urban areas. However, locations inside protected areas tend to have greater biomass (Fig. 2; Supplementary Table 2).

### Anthropogenic associations with food-web persistence

Beyond the above effects of human activity on the size of the food webs, we now turn to the anthropogenic associations with the persistence of the food web as colour coded in Fig. 1. Specifically, our results show that fishing effort, human density and TSA are associated with a decrease in community persistence (Fig. 2). The conservation status of a site, on the other hand, is positively associated with food-web persistence (Fig. 2). Importantly, these correlations between food-web persistence and anthropogenic influences remain significant even when total biomass is accounted for (Fig. 2). This shows that anthropogenic effects on food-web persistence are beyond any signal in total biomass.

### Role of food-web structure

Some of the above associations between anthropogenic effects and food-web persistence are partly explained by human-induced changes in food-web structure and biomass. For example, we found that communities surrounded by higher human densities have a lower tendency to be organized in compartments (Fig. 2; Supplementary Table 4). In turn, consistent with theory^21^, our results show that food-web compartmentalization is associated with an increase in persistence (Supplementary Table 1). Therefore, the negative association between human density and food-web persistence can be explained by a decrease in food-web compartmentalization. Since we control for the number of species, the change in food-web structure is driven by a change in the identity of the constituent species.

### Role of food-web biomass

Part of the association between fishing effort and food-web persistence is explained by the negative association between fishing effort and total community biomass (Supplementary Table 2). More intensively fished communities tend to have a significantly lower fish biomass. However, it is not the reduction of the overall community biomass, but the decrease in the biomass of certain species targeted by fishing^2^, that provides a highly plausible mechanism explaining the relationship between fishing effort and community persistence^16^. The effect of fishing effort on community biomass reveals that the reduction of the targeted species' biomass is not compensated by an increase in some other species biomass. Therefore, fishing is correlated with an overall decrease in community biomass.

Similarly, those areas suffering higher TSAs tend to have lower community biomass (Supplementary Table 2). A lower community biomass, in turn, is related to a lower food-web persistence (Fig. 2). Since TSA is related with climate change, this represents another pathway in which anthropogenic stressors are negatively associated with a decrease in food-web persistence.

Last, our analysis shows that sites located inside protected areas harbour a significant larger community biomass (Supplementary Table 2) than those outside (as reported by refs 28, 29), which in turn is positively correlated with food-web persistence (Fig. 2). This results on the observed association between the conservation status of the site and food-web persistence. Further analyses show that while there is no association between marine protected area (MPA) age and food-web persistence, there is a significant positive association of MPA size with persistence (effect=0.068; s.e.=0.020; *df*=245; *t*=3.425; *P*<0.001).

## Discussion

We have mapped community persistence along a wide regional scale similar to how ecologists plot maps of species richness. While the local number of species is informative of the ecological value of a site, our approach goes a step further by looking at the ability of these communities to persist and withstand future perturbations. Although we cannot infer causality from our statistical analysis, we provide strong evidence revealing a new dimension of the wide-scale impact of human activities in the biosphere. Thus, human activity may not only be affecting population abundances of independent species, but it may also be eroding the robustness of these food webs and therefore their ability to persist in the future.

Availability of spatially distributed information on food-web structure will open the door towards more functional, dynamically based resource management and conservation. If we want to conserve not only species but the persistence of entire communities and the services they provide, we need to ensure the maintenance of trophic structures.

## Methods

### Food webs

The Caribbean food web^16^ describes the trophic relationships between 249 species/trophic groups in a wide geographic region of the Caribbean sea, comprising ∼1,000 km^2^ and stretching from the surface to 100-m depth. Obtained through gut content analysis^17^,^18^, it is the most complete and accurate description of the feeding relationships in the region. One limitation that should be noted is that the level of resolution of the food web is not homogeneous through all ecological groups. Thus, while fishes are resolved at the species level, other species such as zooplankton and invertebrates are highly aggregated. It should also be acknowledged that sampling surveys may fail to account for cryptic species and/or small species difficult to identify while in the field across a vast region (see Supplementary Information of ref. 16 for a detailed description of the strengths and limitation of data). Regardless of the nature of these limitations, they do not systematically vary among sites so as to be likely to generate spurious results. Another limitation is represented by our lack of information on diet shits. It has been shown theoretically^30^,^31^ that interaction plasticity increases persistence. We do not know, however, if interaction plasticity is constant across sites. Therefore, we decided not to consider diet shifts and to use a model that is more grounded in the food web modelling tradition. Also, although we could have included diet shifts from a theoretical perspective (assigning a certain probability), the number of free parameters would have increased enormously, precluding any general conclusion. From all possible scenarios, our results would only become weaker if less persistent food webs could become more plastic and therefore experience a higher increase in persistence.

The Atlantic and Gulf Rapid Reef Assessment (AGRRA)^32^ is a multinational consortium of scientists in the Caribbean Region monitoring the status of coral reefs in a standardized manner. Under this methodology, a highly precise database has been compiled. All scientists involved have higher education degrees and specializations in the study of coral reefs as to ensure data consistency and reductions in human errors implementing the sampling and identifying species. It contains presence–absence information, along with the biomass (g m^−2^), size and number of individuals—per square metre of sampling area—of the fish species found at 701 different locations across the region (Fig. 1). The sampled units were underwater visual censuses over an area of 50 m^2^. The number of sampling units ranged from 6 to 10 per site. Additional details can be found at www.agrra.org.

By combining both databases, we obtain not only the potential food web at every location (Supplementary Fig. 1), but also the body mass of the individuals, and the strength of the interactions. The survey does not provide data on primary resources. However, we know which type of primary producer is targeted by each herbivore species. To ‘feed' the herbivores, we introduce three primary producers in the networks: benthic autotrophs, plankton and colonial invertebrates. Regarding secondary consumers, it may happen that a few of them have been sampled in a site, but none of their prey has been found in that site. In those cases, we add a node called ‘alloctonous input' that simulates the pool of prey for those secondary consumers^16^ (Supplementary Fig. 1). The result of this procedure is a set of 701 local networks of sizes ranging from 15 to 37 species, and connectances from 0.04 to 0.16.

### Dynamic bioenergetic model

To simulate species dynamics, we use the bioenergetic model proposed by Yodzis and Innes^33^ and widely used in recent years^16^,^19^,^20^,^34^. This model simulates the biomass *B* of each species over time. The change in biomass is a function of growth, respiration, and biomass gain and loss trough predation. Mathematically, for species *i*, the model can be written as:


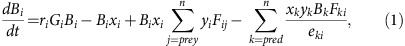


where *G*_*i*_ is the normalized growth rate of basal species; *r*_*i*_ is the mass-specific maximum growth rate; *x*_*i*_ is the mass-specific metabolic rate; *y*_*i*_ is the species maximum consumption relative to its metabolic rate; *e*_*ki*_ is the fraction of the biomass of species *i* lost due to consumption by species *k* that is actually metabolized; *F*_*ij*_ is the type II functional response described as:


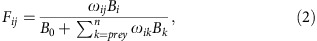


where *ω*_*ij*_ is the interaction preference between species *i* and *j* measured as the relative fraction of prey species *i* in the gut content of predator *j*, and *B*_0_ is the half-saturation density. For basal species, *x*_*i*_=0, while for consumers *G*_*i*_=0. Interaction strength depends on preference and on the biomass of the species involved in the interaction^16^.

The growth rate *G*_*i*_ of basal species is calculated as a competition model:


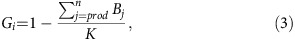


where *K* is the carrying capacity, and for every location it is equal to the biomass of the most abundant species in that location *prod* indicates the total number of primary producers.

The timescale of the system is defined by normalizing the mass-specific growth rate of a basal species used as reference to one. Then, we also normalize the mass-specific metabolic rate *x*_*i*_ and the maximum consumption rate relative to its metabolic rate *y*_*i*_ by the metabolic rates. Therefore, the mass-specific maximum growth rate *r*_*i*_ is equal to one, and *x*_*i*_ is defined as follows:


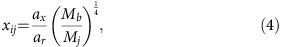


where *a*_*r*_ is the maximal production-to-biomass ratio for ectotermic vertebrates, with *a*_*r*_=*a*_*y*_−*a*_*x*_. The allometric constants *a*_*y*_ and *a*_*x*_ are, respectively, defined as the maximal ingestion and respiration rates for ectothermic vertebrates. *M*_*j*_ is the average body mass of the predator and *M*_*b*_ is the body mass of a reference basal species (set to 1). Finally, the species maximum consumption rate relative to its metabolic rate is defined as 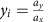.

Average body mass of prey and predators are obtained empirically trough the AGRRA survey and might be different at every location. The allometric constants for ectotermic vertebrates take the values *a*_*y*_=8.9 and *a*_*x*_=2.3. The half-saturation density takes the value *B*_0_=0.75. The fraction of the biomass of prey lost to consumption by the predator that is actually metabolized (*e*_*ij*_) is 0.85. The initial biomasses at each location are those described by the AGRRA survey and are measured in g m^−2^.

The unknown preferences of the interactions between primary producers and herbivores, and between the alloctonous input and the fishes that feed on it are sampled from a probability distribution. To obtain this distribution, we pool together all the known interaction preferences in the local food webs and then fit every possible distribution to the data. The best fit is to a log-normal distribution of *μ*=−4.14±0.10 and *σ*=1.09±0.07.

The unknown initial biomass of the basal species and the alloctonous input are also taken at random from a distribution. To parametrize the distribution, we have used the biomass of each species at each location. The best fit is to a log-normal distribution of *μ*=2.72±0.02 and *σ*=2.27±0.01. With this procedure, even those parameters that are not directly measured in the field are taken from distributions fitted to what is observed in the field.

To solve the system of equations, we use the Runge–Kutta method implemented in Matlab under function ode45. To avoid numerical inconsistencies, we do not allow the solution to have negative biomass values; and to improve accuracy, we manually decrease the relative error tolerance down to 10^−6^. We let the model run for a thousand time steps—enough time for the model to reach the steady state. Since some parameters are not directly observed in the field and are taken from the parametrized distributions, we run a hundred replicates taking those parameters at random from their distributions. Although most studies of this kind run a larger number of replicates, the precise parametrization of the local food webs allows us to obtain a convergent value of average persistence even after 20 replicates.

### Compartmentalization analysis

The modularity *M* of a network is defined as the fraction of links within compartments minus the expected fraction of such links^35^. The expected fraction is estimated from a randomization of the network where nodes have the same number of interactions but they are rewired to other nodes. In a directed random network, the probability of finding a link between two vertices *i* and *j* is 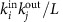, where 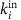 is the in-degree of the node *i*, 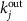 is the out-degree of node *j*, and *L* is the number of links in the network. Therefore, the modularity of directed networks can be defined as:


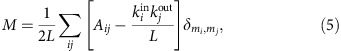


where *A* is the adjacency matrix of the network, *δ*_*ij*_ is the Kronecker delta symbol, and *m*_*i*_ is the label of the module to which the node *i* is assigned. The maximum value of *M*, obtained with the algorithm described in ref. 36, is the modularity of the network.

### Socioeconomic impacts and Geographic Information Systems analysis

Fishing effort is measured as tons of fish per km^2^ per year, as taken from Halpern *et al*.^37^ It is an aggregated measure of different fishing practices (artisanal, demersal and pelagic). The fishing effort at each location is therefore calculated as the intersection between the sampling site coordinates and the fishing effort raster layer.

The number of human inhabitants near the reef is obtained from the Gridded Population of the World Database^38^. To calculate the human population size around every sampling site, we establish a 25-km radius^39^ around the coordinates of the sampling site and then count the number of humans within the radius. This choice was made by Mora *et al*.^39^ based on a tradeoff between the resolution of the data and the buffer over which human populations affect coral reefs directly.

The delimitation of the MPAs in the Caribbean Sea is obtained from a global database of MPAs (WDPA; www.protectedplanet.net). The overlap between the protected areas and the sampling site coordinates determines whether a sampling site is inside a MPA.

TSA is defined as the weekly sea surface temperature (SST) minus the maximum weekly climatological SST. It is obtained from remote sensing data downloaded from the NOAA (National Oceanic and Atmospheric Administration). In particular, we used data from the Coral Reef Temperature Anomaly Database. The database uses Pathfinder Version 5.2 SSTs to quantify thermal stress patterns on the world's coral reefs between November 1981 and 2010.

### Statistical analysis

From each of the 701 sampling sites, we have information on the following variables: simulated persistence, fishing effort, human density, species richness, network connectance, network modularity, geographical coordinates, total recorded biomass, TSA, and finally whether that location is located within a MPA. However, it is unlikely that these variables explain the totality of the observed variance. To incorporate unknown variables that may affect network structure and dynamics, we use a linear mixed effect model that assigns a different intercept for each sampling site.

To model logit(persistence), we use as a random factor the sampling site's ID, acknowledging in this way intrinsic differences within sites. As fixed effects, we use the log of fishing effort, the log of human density, species richness, network modularity, latitude, longitude, protected areas as a categorical factor with two levels (protected or unprotected), and log of recorded total biomass. We excluded other variables—such as phylogenetic diversity and network connectance—that were strongly correlated with any of the variables used as fixed effects. All variables are scaled so variance is of the order of one. The model is fitted by maximizing the log-likelihood. The output of the linear mixed effect model used to analyse the data are given in Supplementary Table 1.

To construct a linear mixed effect model for community biomass, we use as a random factor the different sites and as fixed effects the log fishing effort, the log of human density, food-web modularity, geographical coordinates, the number of species in a certain site, TSA at the site and the conservation status of the site—as a categorical factor with two levels (protected or unprotected). All variables are scaled so variance is of the order of one. The model is fitted by maximizing the log-likelihood. Details of the statistical results are given in Supplementary Table 2.

We also explored the variables that have an effect on the current biodiversity of the sampling sites. To model species richness, we use as a random factor the different sites, and as fixed effects the log fishing effort, the log of human density, modularity, geographical coordinates, the TSA, community biomass, the conservation status of the site as a categorical factor with two levels and log of recorded total biomass in a certain site. All variables are scaled so variance is of the order of one. The model is fitted by maximizing the log-likelihood. Details of the output provided by the linear mixed effect model after model selection are given in Supplementary Table 3.

To study the factors that affect food-web structure, quantified as modularity, we use as a random factor the different sites, and as fixed effects the log fishing effort, the log of human density, number of species, geographical coordinates, the TSA, community biomass, the conservation status of the site as a categorical factor with two levels and log of recorded total biomass in a certain site. All variables are scaled so variance is of the order of one. The model is fitted by maximizing the log-likelihood. Details of the output provided by the linear mixed effect model after model selection are given in Supplementary Table 4.

So far, we were considering whether a site is inside or outside a MPA. But we could also analyse which properties of the MPA might be driving the observed effect. To do so, we performed a linear mixed effect model analysis similar to the one underlying Fig. 2 of the main text—with persistence as the response variable—but considering only the sample sites located within a protected area. This allows us to add MPA size and age as predictive variables, and not just whether a site is inside or outside a MPA.

In all cases, we performed a model selection to obtain the simplest model with the strongest explanatory power. We first run a linear mixed effect model with all the possible variables and obtain the effect sizes and the *P* values for each of the variables. From this first model, we remove the variable with the highest *P* value. Then, we run another linear mixed effect model without that variable. We iterate this process until all remaining variables are significant. We follow this procedure for all the statistical models involved.

## Additional information

**How to cite this article**: Gilarranz, L. J. *et al*. Anthropogenic effects are associated with a lower persistence of marine food webs. *Nat. Commun.* 7:10737 doi: 10.1038/ncomms10737 (2016).

## Supplementary Material

Supplementary Information Supplementary Figure 1 and Supplementary Tables 1-4.
