# Supplementary material for: Anthropogenic effects are associated with a lower persistence of marine food webs
Source: Nat Commun. 2016 Feb 12;7:10737. doi: 10.1038/ncomms10737 (PMC4754348; doi:10.1038/ncomms10737)
Supplement: Supplementary Information — Supplementary Figure 1 and Supplementary Tables 1-4. [file ncomms10737-s1.pdf]

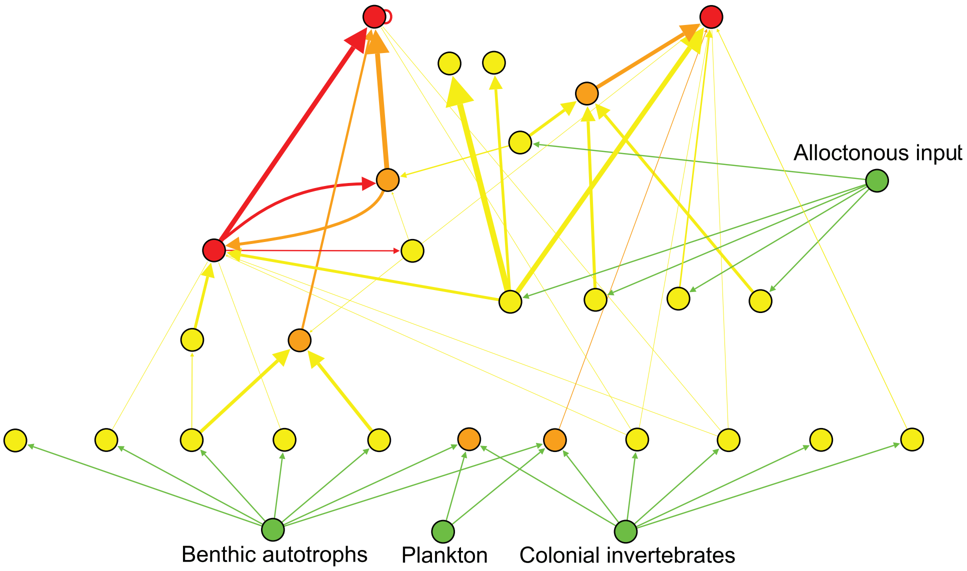

Supplementary Figure 1 | Feeding the herbivores. This represents a subset of one of the local food webs. Node color indicates the number of preys a species has. Species depicted in red feed on a higher number of food sources. Resources are depicted in green. The AGRRA survey classifies herbivore species in three different groups: benthic autotrophs, plankton, and colonial invertebrates. We should therefore connect the food webs to those three different groups. Also, for some predator species we know they are present but none of their prey species has been sampled in that location. We link those predators with an alloctonous input.

Supplementary Table 1 | Results of the linear mixed-effect model used to explore factors correlated to food-web persistence.

	Effect	Std.Error	DF	t-value	p-value
(Intercept)	-0.665	0.013	545	-49.404	< 0.001
Fishing effort	-0.029	0.013	545	-2.179	0.030
Modularity	0.120	0.015	545	7.893	< 0.001
Number of species	-0.310	0.017	545	-18.255	< 0.001
Longitude	-0.058	0.014	545	-4.293	< 0.001
Total biomass	0.042	0.016	545	2.658	0.008

Supplementary Table 2 | Results of the linear mixed-effect model used to explore factors correlated to the local observed biomass.

	Effect	Std.Error	DF	t-value	p-value
(Intercept)	-0.333	0.033	619	-10.036	< 0.001
Fishing effort	-0.103	0.024	619	-4.220	< 0.001
Longitude	0.262	0.025	619	10.500	< 0.001
Thermal stress anomaly	-0.118	0.026	619	-4.578	< 0.001
Protected areas	0.247	0.048	619	5.108	< 0.001
Number of species	0.419	0.024	619	17.284	< 0.001

Supplementary Table 3 | Results of the linear mixed-effect model used to explore factors correlated to the observed number of species.

	Effect	Std.Error	DF	t-value	p-value
(Intercept)	-0.069	0.042	696	-1.642	0.101
Human density	-0.118	0.031	696	-3.867	< 0.001
Modularity	0.394	0.031	696	12.767	< 0.001
Protected areas	0.142	0.061	696	2.322	0.021
Total biomass	0.346	0.031	696	11.177	< 0.001

Supplementary Table 4 | Results of the linear mixed-effect model used to explore factors correlated to food-web modularity.

	Effect	Std.Error	DF	t-value	p-value
(Intercept)	0.163	0.025	649	6.598	< 0.001
Human density	-0.098	0.026	649	-3.796	< 0.001
Number of species	0.267	0.028	649	9.714	< 0.001
